# A nomogram for identifying premyopia and myopia candidates in Chinese children: focusing on those with cycloplegic spherical equivalent refraction ≤ + 0.75D

**DOI:** 10.1186/s12886-025-04278-3

**Published:** 2025-08-28

**Authors:** Jing Wu, Cong Zhang, Jingying Wang

**Affiliations:** 1 Ophthalmology Department, The University of Hong Kong-Shenzhen Hospital, ShenZhen, Guangdong China; 2 Ophthalmology Department, The Shapingba Hospital, Chongqing University, Peoples Hospital of Shapingba District, Chonqing, China; 3 Medical Technology Department, Chongqing Medical and Pharmaceutical College, Chonqing, China

**Keywords:** Premyopia, Nomogram, Primary refractive error screening, Myopia

## Abstract

**Background:**

Primary refractive error screening parameters are commonly employed in clinical and community settings before cycloplegic assessment of myopia, however, their utility in identifying premyopia and myopia intervention candidates remains underexplored. This study aimed to develop a nomogram based on these routinely measured parameters to support clinical decision-making for premyopia and myopia prevention.

**Methods:**

Pediatric patients (aged 4–17 years) from two medical centers in China were enrolled in this retrospective cohort study. A predictive model for the candidates of premyopia and myopia intervention was developed using logistic regression with multiple imputations. The model included the following primary screening parameters: age, gender, uncorrected visual acuity (UCVA), average corneal curvature (ACC), non-cycloplegic spherical equivalent refraction (NCSER), axial length (AL), and the axial length to average corneal radius of curvature (AL/ACRC) ratio. The efficacy of the model was assessed using the area under the receiver operating characteristic (ROC) curve, calibration curves, and decision curve analysis (DCA). R was employed to conduct all statistical analyses.

**Results:**

A total of 1006 participants (507 females, 499 boys) were enrolled, with 87.4% demonstrating cycloplegic spherical equivalent refraction (CSER) ≤ + 0.75D. In multivariate logistic regression, UCVA, NCSER, AL, and AL/ACRC were identified as independent predictors. These predictors were incorporated into a nomogram to predict the candidates for premyopia and myopia intervention. The nomogram exhibited exceptional discrimination in the derivation set (AUC = 0.971, 95% CI: 0.957–0.984), whereas in the external validation set, the AUC was 0.921 (95% CI: 0.866–0.976) when a cutoff of 0.851 in derivation set was employed. Calibration was verified through the calibration curve and Hosmer-Lemeshow tests (*P* = 0.99 and *P* = 0.96, respectively), and the decision curve analysis demonstrated robust clinical utility for threshold probabilities of 0.10–1.00 in the derivation set and 0.20–1.00 in the external validation set.

**Conclusion:**

The nomogram derived from the parameters of primary refractive error screening has the potential to preliminarily predict premyopia and myopia intervention candidates, thereby facilitating clinical decision-making in the context of premyopia and myopia prevention.

## Background

Myopia is among the most prevalent public health issues globally [1]. In East Asia and Singapore, the prevalence of myopia ranges from 80–90% [2]. This high incidence is concerning, especially considering that early onset myopia significantly increases the likelihood of developing high myopia [3]. High myopia, in turn, markedly elevates the risk of permanent vision-threatening ocular disorders, such as myopic macular degeneration, glaucoma, and cataract [4]. Given the significant prevalence of myopia and the serious consequences it can entail, early implementation of effective prevention strategies is essential.

Premyopia is the most prevalent refractive status in Chinese children, accounting for 52% of the population [5]. It is a refractive state in children where a combination of baseline refraction, age, and other quantifiable risk factors provide a sufficient likelihood of the future development of myopia to merit preventative interventions [6–9]. This pre-pathological state warrants preventive intervention due to its high conversion probability to myopia. As myopia progression proves clinically recalcitrant once established [5], targeted intervention in premyopic children is critical for either maintaining emmetropization or delaying myopia onset [10]. Practically, myopia intervention has been conducted on premyopic children whose refractive state ≤ + 0.75 D but > − 0.50 D in previous studies [11–13].

Consequently, based on the International Myopia Institute’s diagnostic criteria, where myopia is defined as refractive state ≤ −0.50 D, and premyopia as > −0.50 D to ≤ + 0.75 D with concomitant risk factors [14], coupled with education’s established role as an unavoidable myopia risk factor in Chinese children [15], this study classified Chinese children presenting with refractive state ≤ + 0.75 D as premyopia and myopia intervention candidates.

In clinics, refractive state ≤ + 0.75D were confirmed by cycloplegic refraction [9]. However, many children are unsuitable for cycloplegia for many reasons, such as side effects like photophobia and tearing; ocular conditions like high ocular pressure, narrow chamber angle, and amblyopia [16]; system disease history like cardiovascular and nervous system disease history; or in some scenarios like eye health screening in schools and communities because of disturbance of studying activity during accommodation paralysis in cycloplegia [17]. Besides, in actual practice, eyecare providers would not regularly consider further cycloplegia in a child with a minor minus (positive) degree in non-cycloplegic spherical equivalent refraction (NCSER) to improve clinical efficiency. Thus, Cycloplegic refraction is not available for all premyopia and myopia intervention candidates in practice.

Primary refractive error screening is commonly performed in eye clinics for candidates for premyopia and myopia interventions. This screening includes visual acuity tests, non-cycloplegic autorefractor tests, and ocular biometer tests. These methods are preferred due to their shorter examination times, non-contact operation, and absence of side effects compared to the golden standard of cycloplegic refraction [17]. According to the Chinese government recommendation, the primary refractive error screening was adopted in schools or communities at least twice yearly for earlier myopia detection. Thus, Age, gender, uncorrected vision acuity (UCVA) by visual acuity test, average corneal curvature (ACC), NCSER by autorefractor test, axial length (AL), the axial length to average corneal radius of curvature (AL/ACRC) by ocular biometer test were routinely gained by eyecare providers without cycloplegia. These parameters were broadly used in the myopia prediction model that cycloplegic spherical equivalent refraction (CSER) ≤−0.50 D [18–23]. Nevertheless, only one study has attempted to develop a predictive model specifically for identifying candidates for intervention in premyopia and myopia: the Nanjing cohort study [24]. This study utilized AL, AL/ACRC, and parental myopia as predictors to estimate overall survival at one and two years for individuals with premyopia, but notably, it lacked a external validation step [24]. Consequently, there remains a significant gap in research concerning the development of clinical prediction models for identifying candidates for intervention in premyopia and myopia, particularly those that incorporate validation steps and factors derived from primary refractive error screenings. This study analyzed the primary refractive error screening parameters, including age, gender, UCVA, ACC, NCSER, AL, and AL/ACRC. The objective was to create a nomogram that predicts candidates for premyopia and myopia interventions, validated using external data.

## Methods

### Research design and participants

**Fig. 1:**
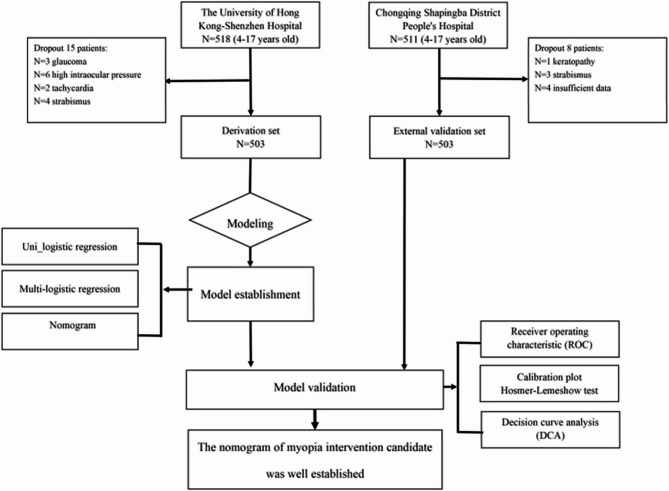
Flow diagram of study design

This retrospective cohort study included patients from 2 distinct medical centers in China. The derivation cohort comprised patients at The University of Hong Kong-Shenzhen Hospital, and the external validation cohort consisted of patients admitted at the Chongqing Shapingba District People’s Hospital. Examining the electronic medical records systems at the Chongqing and Shenzhen ophthalmology clinics, we picked up seven present predictors from primary refractive error screening: gender, age, UCVA, ACC, NCSER, AL, and AL/ACRC. Otherwise, most Chinese children would accept the first myopia screening after 3 years old as the Chinese government demanded, and the children after 18 years would be recognized as adults [25] with little necessity for myopia control. Hence, this study included pediatric patients aged between 4 and 17 years who underwent eye clinic examinations. Between April 2024 and October 2024, the definitive patient population for evaluation comprised 1006 individuals. Figure 1 illustrates a flow diagram of the study design.

Exclusion criteria were: (1) the presence of ocular organic diseases, such as high intraocular pressure, strabismus, keratopathy(keratoconus), cataract, glaucoma, amblyopia, and fundus disease, accommodative spasms which were considered to influence refractive status. (2) the presence of systemic diseases history such as hypertension, autoimmune disease, convulsions, cardiovascular system disorder, central nervous system disorder, which were considered to influence cycloplegic results. (3) the best-corrected monocular visual acuity was below 0.8 for children aged 6 and older, and below 0.5 for children aged 3 to 5 [26]. The participants with the above conditions were meticulously ruled out based on their present diagnoses documented in their medical records.

### Diagnosis criteria of premyopia and myopia intervention candidates

The clinical gold standard for myopia is CSER ≤−0.50D, while premyopia is characterized by CSER >−0.50D and ≤ + 0.75D, accompanied by risk factors like baseline refraction, age or others for myopia [9]. Given the significant prevalence of premyopia (52%) [5] among Chinese children and the unavoidable role of education as a risk factor for myopia in this demographic [15], premyopia represents a refractive condition with limited hyperopic reserves, placing children at an elevated risk for myopia and necessitating preventive interventions. Several studies have implemented myopia interventions—such as repeated low-level red-light therapy and low-concentration atropine—in children with CSER ≤ + 0.75 D but > − 0.50 D [11–13], suggesting that therapeutic intervention may be warranted in not only myopic (CSER ≤ − 0.50 D) but also pre-myopic (CSER ≤ + 0.75 D but > − 0.50 D) pediatric populations. Consequently, this investigation identified Chinese children with CSER ≤ + 0.75D as candidates for premyopia and myopia intervention.

#### Ethics approval

This study adhered to the Declaration of Helsinki and the Transparent Reporting of a Multivariable Prediction Model for Individual Prognosis or Diagnosis guidelines. The medical ethics committees of the University of Hong Kong-Shenzhen Hospital (approval number: [2024]156) and the Chongqing Shapingba District People’s Hospital (approval number: 202309) approved this study protocol. Because the study is retrospective and does not involve any risk, the requirement for informed consent was waived.

### Statistical analysis

During the data preprocessing step, the spherical equivalent refraction (SER) was calculated by summing half of the cylinder power with the sphere power. At the same time, the anterior corneal curvature (ACC) was derived as the average of the horizontal and vertical meridians of corneal curvature. The ACRC was computed using the formula ACRC = 337.5/ACC, and the AL/ACRC ratio was derived as the ratio of AL to ACRC. We chose the right eye for the final analysis due to the significant link between the eyes.

Statistical analysis was conducted utilizing R software (version 4.4.2; R Foundation for Statistical Computing, Vienna, Austria). First, Missing data was treated as missing at random for the complete data in our study. Multiple imputation via chained equations was employed for the limited missing data (missing data by cases = 7.06% < 10%), creating a single imputed dataset using the R *mice* package for subsequent analysis. The R *compareGroups*, *glue*,* tidyr*, and *broom* packages were used to describe the character of our research cohort. Subsequently, we used R. *cat* to conduct collinear diagnosis on the variables that showed a significance level of *P* < 0.05 in univariate logistic regression analysis. Following this, we employed the *rms* package in R to conduct multiple logistic regression with variables showing a significance level of *P* < 0.05 in univariate logistic regression and passed collinearity diagnosis. In the predictor selection process, variables that showed a significance level of *P* < 0.05 in univariate logistic regression and passed collinearity diagnostics were retained for further analysis through multiple logistic regression (AL/ACRC was changed in AL/ACRC*100 for decreasing odds ratio). Using a bidirectional stepwise approach guided by the Akaike Information Criterion (AIC) in the multiple logistic regression [27], the important predictors for identifying candidates for premyopia and myopia intervention were identified. Finally, a point-based nomogram was constructed to visualize the logistic regression model based on the important predictors for identifying candidates for premyopia and myopia. This was achieved using the R. *regplot* package, which converted the regression coefficients of the final predictors (selected through bidirectional stepwise approach) into a quantitative scoring system. Specifically, each predictor’s contribution was scaled to a 0–100-point range based on its relative weight in the model. The total points for a given patient are summed and aligned to a probability axis, enabling direct clinical risk estimation. Otherwise, we carried out a sensitivity analysis that included all patients without the multiple imputation and missing values were deleted to reconfirm the entry variables of the model.

Additionally, various validation techniques were employed to assess the accuracy of the risk prediction model in both the derivation and external validation sets. We utilized the R. *pROC* package for the receiver characteristic curve (ROC) analysis [27]. Furthermore, the cutoff point of the nomogram established in the derivation set was utilized as a binary variable in the external validation set for ROC analysis to evaluate the efficacy of the nomogram in identifying candidates for premyopia and myopia intervention. We utilized the R. *rms* package to construct and compute the calibration curves, which were employed to assess the nomogram’s calibration, supplemented by the Hosmer-Lemeshow test using the HLtest.R resource. We utilized the DCA.R resource for decision curve analysis to assess the clinical feasibility of nomograms based on net benefit across various threshold probabilities in the cohort [28].

## Results

### Characteristics of the study cohort

Among the 1006 participants in our analysis, 507(50.4%) were girls, 499 (49.6%) were boys, 745(74.06%) had myopia (CSER≤−0.50D),127 (12.6%) had CSER > + 0.75D, while 879(87.4%) had CSER ≤ + 0.75D. 503 participants (CSER ≤ + 0.75D:82.3%) from the Shenzhen cohort constituted the derivation set, whereas 503 participants (CSER ≤ + 0.75D: 92.4%) from the Chongqing cohort formed the external validation set. Table 1 presents the characteristics of the patients in both groups.

There was no statistical significance in gender and ACC between CSER > + 0.75D and CSER ≤ + 0.75D. Additionally, the derivation set (*N* = 503) and an external validation set (*N* = 503) maintain comparable gender (*P* = 1.000) and ACC(*P* = 0.372) distributions. Nonetheless, additional baseline parameters such as Age, UCVA, NCSER, CSER, AL, and AL/ACRC underscore significant disparities between the derivation set and the external validation set: In comparison to the derivation set, the external validation set comprised an older demographic (median age 11.0 vs. median age 9) with inferior UCVA (median 0.30 vs. median 0.40), more NCSER (median − 1.75D vs. median − 1.25D), more CSER (median − 1.50D vs. median − 1.25D), increased AL (median 24.3 mm vs. median 24.0 mm), and a higher AL/ACRC (median 3.11 vs. median 3.08).

**Table 1:** Demographic characteristics

	ALL	CSER>+0.75D	CSER≤+0.75D	*P* value	Derivation set	External validation set	*P* value
	*N*= 1006(74.06% myopia)	*N*= 127 (12.6%)	*N*=879(87.4%)		*N*=503	*N*=503	
Gender				0.925			1
Girls	507 (50.4%)	65 (51.2%)	442 (50.3%)		253 (50.3%)	254 (50.5%)	
Boys	499 (49.6%)	62 (48.8%)	437 (49.7%)		250 (49.7%)	249 (49.5%)	
Age(years)	10.0 [8.00;12.0]	6.00 [5.00;8.00]	10.0 [9.00;12.0]	<0.001	9.00 [7.00;11.0]	11.0 [9.00;13.0]	<0.001
UCVA	0.30 [0.20;0.60]	0.70 [0.50;0.90]	0.30 [0. 15;0.50]	<0.001	0.40 [0.20;0.60]	0.30 [0. 15;0.50]	<0.001
ACC(D)	43.2 [42.2;44.2]	43.2 [42.2;44.2]	43.2 [42.3;44.2]	0.479	43.2 [42.2;44.2]	43.4 [42.4;44.2]	0.372
NCSER(D)	−1.50 [−2.75; −0.50]	0.50 [0.00;1. 13]	−1.75 [−3.00; −1.00]	<0.001	−1.25 [−2.50; −0. 13]	−1.75 [−2.88; −1.00]	<0.001
CSER(D)	−1.25 [−2.50; −0.38]	1.75 [1.25;2.44]	−1.50 [−2.75; −0.75]	<0.001	−1.25 [−2.38;0. 13]	−1.50 [−2.75; −0.75]	<0.001
AL (mm)	24.1 [23.3;24.9]	22.3 [21.7;22.9]	24.3 [23.6;25.0]	<0.001	24.0 [23. 1;24.7]	24.3 [23.6;25.0]	<0.001
AL/ACRC	3.09 [3.01;3. 18]	2.87 [2.80;2.91]	3.12 [3.05;3. 19]	<0.001	3.08 [2.96;3. 17]	3.11 [3.05;3. 19]	<0.001

UCVA: uncorrected visual acuity; ACC: average corneal curvature; NCSER: non-cycloplegic spherical equivalent refraction

CSER: cycloplegic spherical equivalent refraction; AL: axial length; ACRC: average corneal radius of curvature; AL/ACRC: axial length to average corneal radius of curvature; D, diopter

### A predictive model for premyopia and myopia intervention candidates

**Fig. 2:**
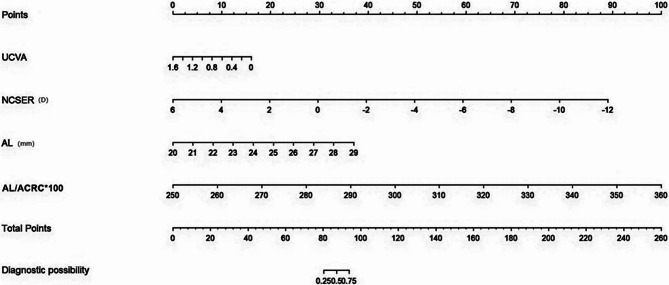
Nomogram to predict the risk of premyopia and myopia intervention candidates

According to the principle of collinearity diagnosis [29], no collinearity among the variables is included in the multivariate logistic analysis (Table 2). Univariate regression analysis was employed to identify predictive factors from the characteristics listed in Table 3, whereas multiple logistic regression was utilized to develop the predictive model. As Table 3 showed, UCVA (OR = 0.2; 95%CI = 0.03–1.17), NCSER (OR = 0.45;95%CI = 0.25–0.8), AL (OR = 1.95;95%CI = 1.13–3.36), and AL/ACRC*100 (OR = 1.16;95%CI = 1.08–1.24) were included in the predictive model. The logistic analysis included all patients without the multiple imputations that missing values were deleted, showing that the variables in the model did not change substantially and that UCVA, NCSER, AL, and AL/ACRC*100 were highly correlated with premyopia and myopia intervention candidates (Table 4). The prediction model utilizing UCVA, NCSER, AL, and AL/ACRC*100 was formulated as a nomogram to assess the risk probability for premyopia and myopia intervention candidates (Fig. 2). For example, employing the nomogram model, a patient with UCVA of 0.6 (about 10 points), NCSER of −1.00D (about 35 points), AL of 23 mm(about 12.5 points), and AL/ACRC*100 of 290 (about 35 points), whose total points was 92.5 and had an estimated probability of premyopia and myopia intervention candidates above 50%; thus, further cycloplegia or myopia intervention would be deemed appropriate by eyecare providers in clinical practice.

**Table 2:** Collinear diagnosis of the variables (*P* < 0.05 in univariate analysis)

Variables	Variance inflation factor	Tolerance
Age	1.58	0.634
UCVA	1.21	0.823
NCSER(D)	1.2	0.832
AL (mm)	1.54	0.647
AL/ACRC*100	1.36	0.736

UCVA: uncorrected visual acuity; NCSER: non-cycloplegic spherical equivalent refraction; AL: axial length; ACRC: average corneal radius of curvature; AL/ACRC: axial length to average corneal radius of curvature; D, diopter

### The validation of the predictive model for premyopia and myopia candidates

**Fig. 3:**
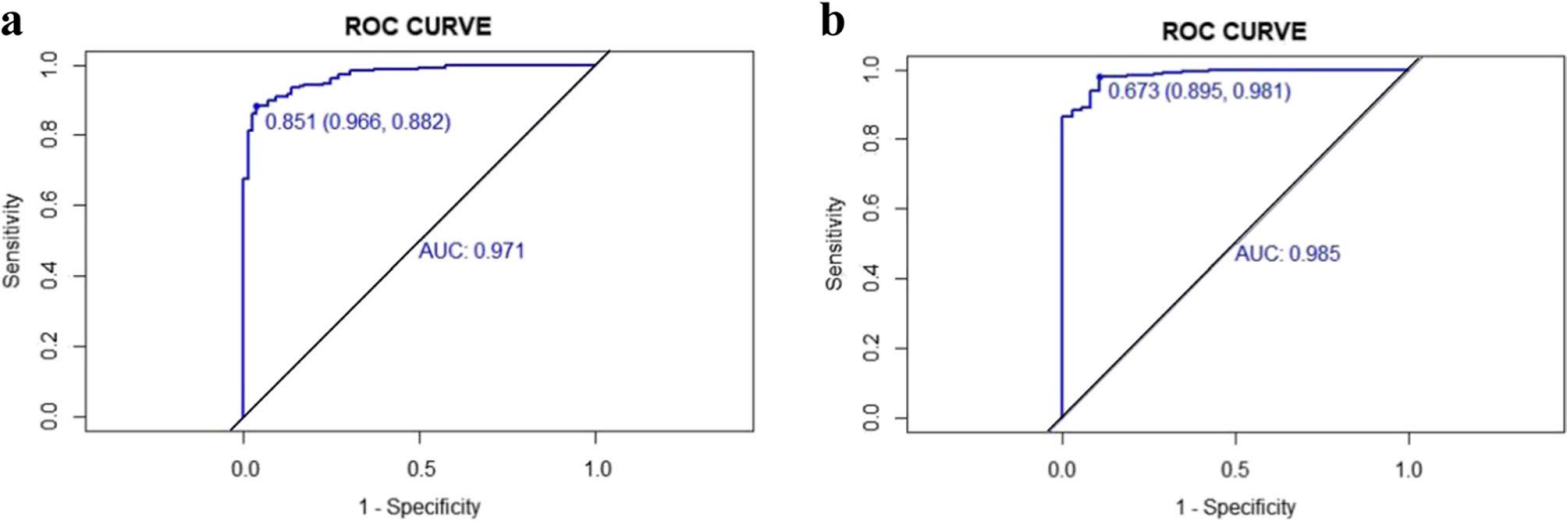
ROC validation of the prediction nomogram for premyopia and myopia intervention candidates. The black line represents the perfomance of the nomogram in the validation set (**a**) and external valiation set (**b**)

For the predictive model, the nomogram’s AUC was 0.971(0.957–0.984) in the derivation set and 0.985 (0.973–0.996) in the external validation set, which indicated good performance (Fig. 3). The nomogram’s cutoff point in the derivation set was 0.851; we used 0.851 as a binary variable in the external validation set and found that the AUC in the external validation set was 0.921(0.866–0.976), which was also acceptable (Table 5).

**Table 3:** Logistic regression analysis of primary refractive error screening parameters for premyopia and myopia intervention candidates

	Predictor variable	Univariate analysis			Multiple analysis (bidirectional stepwise method)		
		OR	95% CI	*P* value	OR	95% CI	*P* value
Premyopia and myopia intervention candidate (CSER ≤+0.75D)	Intercept				0	0-0	0
	Gender	0.81	0.51-1.29	0.379			
	Age (years)	1.76	1.56-2	**<0.001**			
	UCVA	0.01	0-0.03	**<0.001**	0.2	0.03-1.17	**0.074**
	ACC(D)	1.07	0.91-1.25	0.425			
	NCSER(D)	0.14	0.09-0.22	**<0.001**	0.45	0.25-0.8	**0.006**
	AL (mm)	8.56	5.53-13.25	**<0.001**	1.95	1.13-3.36	**0.016**
	AL/ACRC*100	1.27	1.21-1.34	**<0.001**	1.16	1.08-1.24	**<0.001**

UCVA: uncorrected visual acuity; ACC: average corneal curvature; NCSER: non-cycloplegic spherical equivalent refraction

AL: axial length; ACRC: average corneal radius of curvature; AL/ACRC: axial length to average corneal radius of curvature, D:diopter; OR: odd ratio; CI: Confidence Interval; The bolded values represent statistically significant predictors for identifying premyopia and myopia intervention candidates

**Table 4:** Sensitive analysis: logistic regression analysis of primary refractive error screening parameters for premyopia and myopia intervention candidates (data without imputation)

	Predictor variable	Univariate analysis			Multiple analysis (bidirectional stepwise method)		
		OR	95% CI	*P* value	OR	95% CI	*P* value
Premyopia and myopia intervention candidate (CSER ≤+0.75D)	Gender	0.78	0.48-1.27	0.32			
	Age (years)	1.73	1.52-1.97	**<0.001**			
	UCVA	0.01	0-0.03	**<0.001**	0.12	0.02-0.85	**0.033**
	ACC(D)	1.08	0.91-1.27	0.397			
	NCSER(D)	0.14	0.09-0.23	**<0.001**	0.56	0.32-0.98	**0.043**
	AL (mm)	8.96	5.61-14.32	**<0.001**	2.19	1.22-3.9	**0.008**
	AL/ACRC*100	1.29	1.22-1.36	**<0.001**	1.18	1.1-1.27	**<0.001**

UCVA: uncorrected visual acuity; ACC: average corneal curvature; NCSER: non-cycloplegic spherical equivalent refraction; AL: axial length; ACRC: average corneal radius of curvature; AL/ACRC: axial length to average corneal radius of curvature; D, diopter; OR: odd ratio; CI: Confidence Interval; The bolded values represent statistically significant predictors for identifying premyopia and myopia intervention candidates.

**Table 5:** Performance of the nomogram in predicting premyopia and myopia intervention candidates

AUC		95%CI	Best threshold	Specificity (%)	Sensitivity (%)	Accuracy (%)	PPV (%)	NPV (%)	PLR	NLR
Derivation set	0.971	0.957–0.984	0.851	99.6	88.2	89.7	99.2	63.7	26.16	0.12
External validation set	0.921	0.866–0.976	0.5	92.1	92	92	99.3	48.6	11.66	0.09

AUC: area under the curve; 95%CI: 95% confidence interval; PPV: positive predictive value; NPV: negative predictive value; PLR: positive likelihood ratio; NLR: negative likelihood ratio

**Fig. 4:**
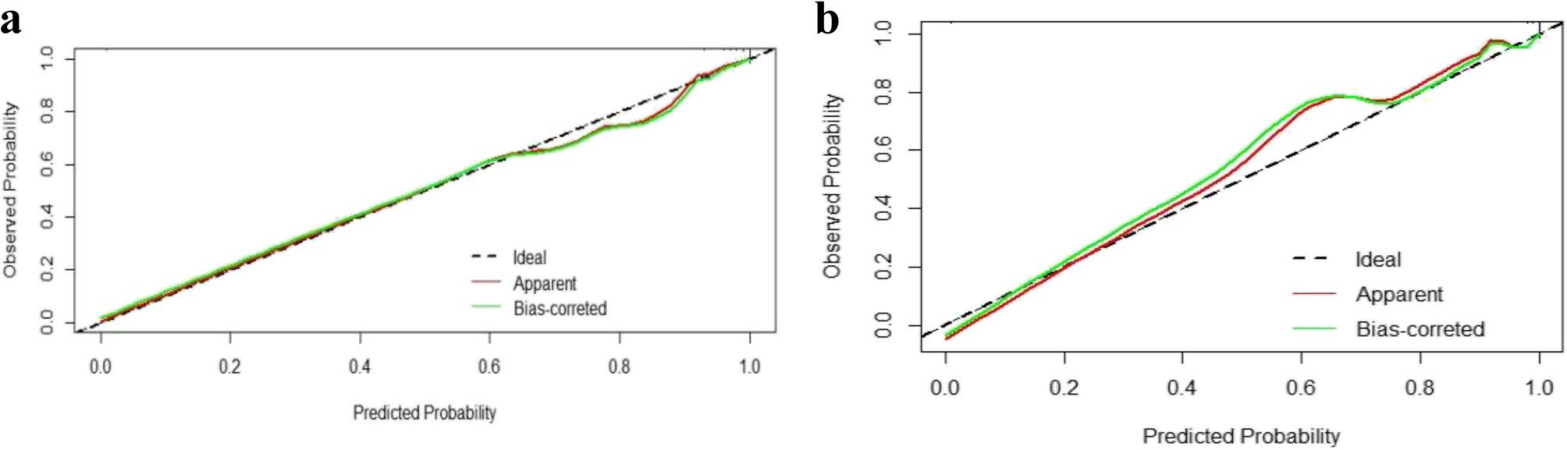
Calibration curve of the prediction nomogram for premyopia and myopia intervention candidates. The diagnoal dashed line (black) represents a perfect prediction by an ideal model, the green line represents the performance of the derivation set (a) and the external validation set (b), with results indicating that a closer fit to the diagnoal dashed line represents

**Fig. 5:**
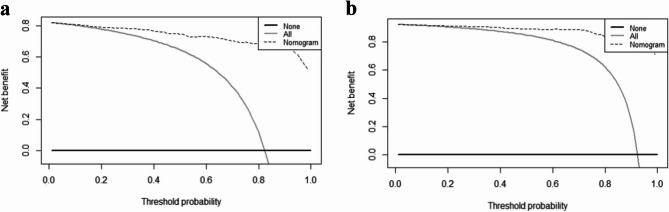
Decision curve analysis for the prediction nomogram for premyopia and myopia intervention candidates. The thick solid line represents the assumption that all participants demand no myopia intervention, the thin solid line represents the assumption that all participants demand myopia intervention, the dotted line represents the risk nomogram, (**a**) from the derivation set and (**b**) from the external validation set

The calibration curves indicated that the predictive model and the validation set exhibited a satisfactory degree of fit (Fig. 4): in the derivation set, the apparent calibration curve showed near-perfect alignment with the ideal reference line throughout the entire predicted probability range (0.0–1.0), with only minor deviations at higher predicted probabilities (> 0.8); in contrast, the external validation set displayed a bias-corrected calibration curve that closely resembled the ideal line in lower-risk strata (0.0–0.5), with deviations observed at predicted probabilities between 0.5 and 0.75. The Hosmer-Lemeshow test indicated a strong consistency between predicted and actual probabilities (derivation set: Chi-square 1.92, *P* = 0.99; external validation set: Chi-square 3.09, *P* = 0.96). The decision curve analysis (DCA) indicated that the model had significant practicality across a broad threshold range (0.10-1.00) in the derivation and 0.20-1.00 in the external validation set (Fig. 5).

## Discussion

This study effectively created and validated a predictive nomogram for assessing the likelihood of premyoia and myopia intervention candidates in Chinese children aged 4–17. By incorporating routinely measured parameters from primary refractive error screening—UCVA, NCSER, AL, and AL/ACRC ratio—the model provides a robust risk assessment tool for premyopia and myopia candidates. The nomogram exhibited robust performance, indicated by a high area under the curve (AUC), well-calibrated plots, non-significant Hosmer-Lemeshow tests, and positive DCA results. These results suggested that the model may serve as a valuable clinical tool for early identification and intervention, ultimately reducing the long-term burden of myopia.

Previous studies on myopia intervention candidates have primarily focused on myopic children [7, 8, 17–19, 21, 24, 30, 31], while recent research has increasingly emphasized early identification and intervention at the premyopia stage [5, 11, 14]. Notably, the only existing nomogram for premyopia and myopia [24] which has not been validated was based on AL, AL/ACRC, and the number of myopic parents, without incorporating primary refractive error screening parameters such as UCVA and NCSER. Among other studies lacking nomogram construction, the findings from the Peking cohort suggested that AL/ACRC could serve as an alternative predictor for identifying hyperopia reserve of premyopia [32]. The data from the Shanghai cohort identified AL/ACRC, UCVA, AL, and NCSER as key risk factors for premyopia and myopia [33]. These studies underscore the value of AL/ACRC, UCVA, AL, and NCSER as strong predictive markers for identifying premyopia and myopia intervention candidates. In this study, we developed a validated nomogram incorporating these routinely measured screening parameters, providing a novel tool for premyopia and myopia intervention.

In the process of validation, the nomogram displayed good discriminative power in both the derivation set (AUC = 0.971, 95% CI: 0.957–0.984) and the external validation set (AUC = 0.921, 95% CI: 0.866–0.976). In contrast to the derivation set, which exhibited a specificity of 99.6% and a sensitivity of 88.2%, indicating potential overfitting and diminished capacity to eliminate false positives in practical scenarios, the external validation set demonstrated a more balanced performance with a specificity of 92.1% and a sensitivity of 92%, thereby preserving exceptional predictive capability and showing robust generalizability. The calibration analysis indicated that the model underestimated risk at probabilities ranging from 0.5 to 0.75 in the validation cohort, perhaps resulting in excessively cautious clinical actions. Notwithstanding the identified biases, the *P* value exceeding 0.05 in our calibration assessments indicated an adequate overall fit, affirming its dependability for clinical applications. The decision curve analysis revealed a superior net benefit compared to both the “None” and “All” strategies across a broad spectrum of threshold probabilities in both the derivation (0.10-1.00) and external validation sets (0.20-1.00), suggesting that the nomogram would provide a significant clinical advantage in identifying premyopia and myopia intervention candidates.

We found no significant correlation between gender, age, or ACC and the identification of premyopia and myopia intervention candidates. This finding aligned with previous studies. The Peking cohort [32] specifically reported no association between ACC and hyperopia reserve in premyopic children, although gender and age were considered. Moreover, In the Nanjing study [24] and the Shanghai cohort [33], gender, ACC, and age were excluded from the multivariate models.

This study’s merits included the external validation of our prediction model utilizing an independent cohort from Shenzhen, characterized by an older demographic and a more severe myopic condition relative to Chongqing. The high accuracy in the external validation set suggested that the nomogram would likely apply to diverse populations across various regions in China. Furthermore, this work developed a nomogram utilizing characteristics from primary refractive error screening often employed by eyecare providers, which may have extensive uses in clinical practice.

Certain limits must also be recognized. Firstly, the current study did not investigate several characteristics that may contribute to premyopia and the identification of candidates for myopia intervention. These include factors such as the myopia status of caregivers [20], caregiver education levels, and lifestyle factors like screen time, educational achievement, and outdoor activity [5]. Additionally, genetic influences and elements related to binocular vision (especially accommodation ability) [26] were not examined. However, these factors were not the primary focus of our research; instead, our objective was to develop a nomogram based on parameters derived from primary refractive error screening, to facilitate clinical use by eyecare providers. Secondly, the nomogram was constructed on a cross-sectional study with retrospective data. Thus, the accuracy of the model we set may be limited, although we use sensitivity analysis to maximize the accuracy of our model. Thirdly, the study cohort included a broad age range (4–17 years), and we recognized that the nomogram may yield different results when subjected to age-stratified analyses although age wasn’t the final predicator enrolled in the final nomogram. Unfortunately, the limited sample size of our study precluded such analyses for predicting premyopia and myopia, which in turn may have affected diagnostic sensitivity and specificity. Finally, future research should involve large-scale longitudinal studies with age-stratified analyses in school-aged children. These studies should incorporate additional predictors, particularly accommodation parameters, to enhance the validity of the nomogram’s clinical application in identifying candidates for premyopia and myopia intervention.

## Conclusions

The developed nomogram, validated externally and based on UCVA, NCSER, AL, and AL/ACRC, showed strong prediction accuracy and practicality. It can be utilized to evaluate individual candidates for premyopia and myopia intervention, hence offering a reference for early intervention decisions on myopia status for eyecare providers.

## Data Availability

The data that support the finding of this study are available from the corresponding author, Jingying Wang, upon reasonable request.This study was approved by The Medical Ethics Committee of the University of Hong Kong-Shenzhen Hospital and the Chongqing Shapingba District People’s Hospital, which waived the requirement for informed consent.

## Abbreviations

UCVA: Uncorrected Visual Acuity
ACC: Average Corneal Curvature
NCSER: Non-cycloplegic Spherical Equivalent Refraction
AL: Axial Length
AL/ACRC: Axial Length to Average Corneal Radius of Curvature Ratio
ROC curve: Receiver Operating Characteristic Curve
DCA: Decision Curve Analysis
